# Nasal Septum Perforation due to Methamphetamine abuse

**Published:** 2013

**Authors:** Mehdi Bakhshaee, Ehsan Khadivi, Masoud Naseri Sadr, Fereshteh Esmatinia

**Affiliations:** 1 *Ear, Nose, Throat, Head and Neck surgery Research Center, Mashhad University of Medical Sciences, Mashhad, Iran.*; 2 *Department of Otorhinolaryngology, Mashhad University of Medical Sciences, Mashhad, Iran.*

**Keywords:** Drug abuse, Methamphetamine, Septal perforation

## Abstract

**Introduction:**

Spontaneous Perforation of the nasal septum is an uncommon condition. Nasal inhalation of substances such as cocaine has long been linked to this Perforation.

**Case Report:**

This report describes the case of a 46-year-old woman who was addicted to methamphetamine and who presented with perforation of the nasal septum.This is the first reported case of nasal septal necrosis linked to nasal inhalation of methamphetamine.

**Conclusions:**

Patient history and assurance regardingillegal drug consumption and abuse is a key point for fast and accurate diagnosis. The pathophysiology of drug-induced sinunasal disease and a review of the literature are also presented.

## Introduction

Perforation of the nasal septum is an uncommon condition. When it occurs, its cause is most often idiopathic or traumatic. Nasal septum perforation may also be the presenting sign of drug addiction or a potentially life-threatening or serious systemic illness, even in an asymptomatic patient (1-5). In this article, we describe a case of nasal septal perforation secondary to methamphetamine abuse. We also briefly review other common causes of perforation of the nasal septum.

## Case Report

A 46-year-old woman presented with nasal congestion, mucopurulent rhinorrhea and nasal obstruction. The symptoms were reported to have started only 3 months earlier. On examination her nasal cavities were filled with necrotic debris and extremely sensitive to touch. In addition, deformity in the form of saddle nose was observed. The soft palate, hard palate, tonsils and posterior oropharyngeal wall were normal. 

**Fig1:**
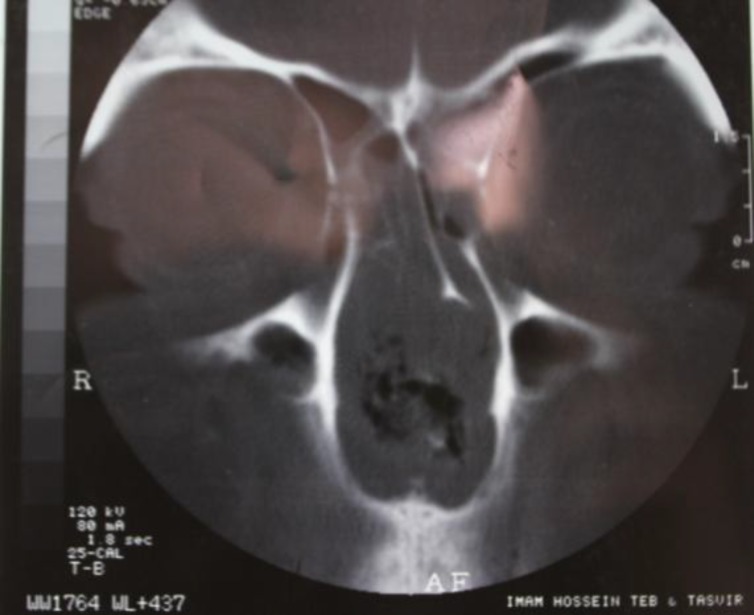
Radiologic view of the patient’s paranasal sinus

The patient initially denied any drug abuse, but reluctantly admitted to habitually snorting a crushed preparation of metham- phetamine for 3 years. A CT scan of the paranasal sinus showed mucoperiostal swelling of all sinuses with necrosis of the nasal septum (Fig 1). The ulcerative process in the patient’s nose was rapidly progressive over the following 4 months.

**Fig2:**
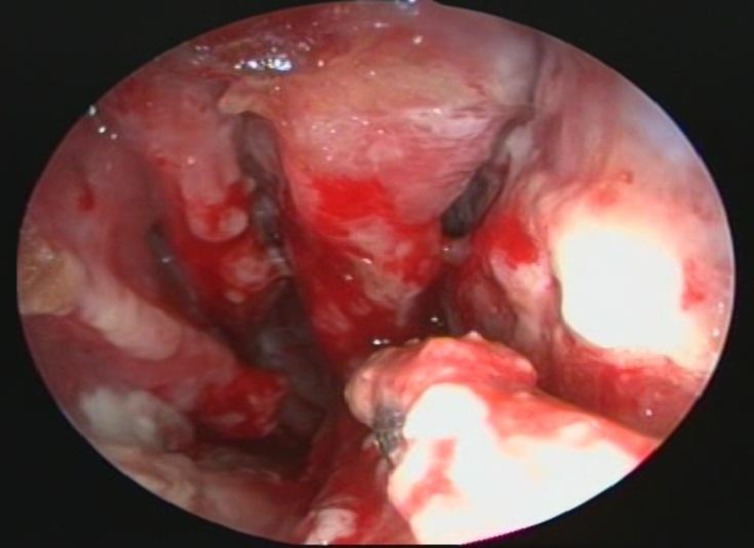
Endoscopic view of the patient’s nasal cavity

The patient’s erythrocyte sedimentation rate was normal and the following tests produced no pathological results: complete blood count, liver and renal function tests, serology for syphilis, bacteriological cultures including stains for fungi and tuberculosis, chest radiograph, extensive urine analysis, examination of rheumatoid factors as well as antinuclear and antimitochondrial tests. In an endoscopic examination a large septal perforation accompanied by excessive necrotic tissues in the nasal space was obvious (Fig 2).

**Fig3:**
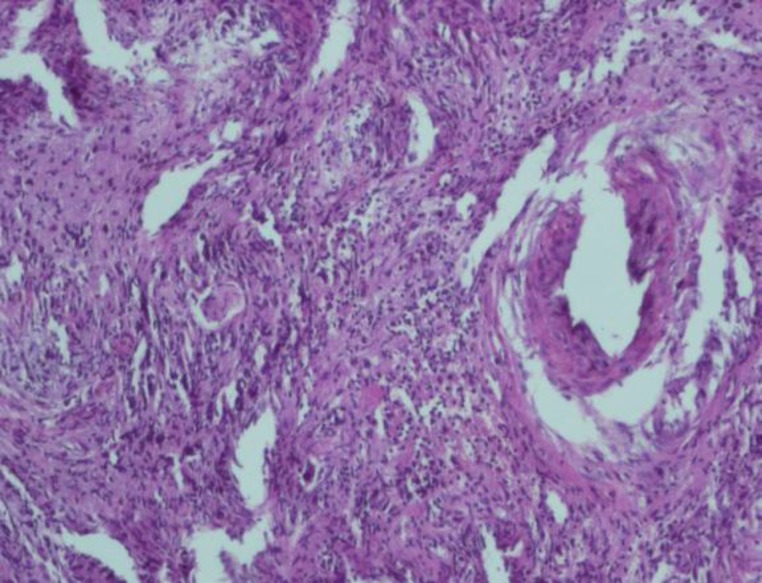
Pathologic view of the patient’s sinonasal mucosa

Multiple biopsies of the nose and palate were taken and showed necrosis and ulceration with signs of inflammation, but no evidence for vacuities or granulomatosis (Fig 3). Further testing for antineutrophil cytoplasmic antibodies (C-ANCA) was negative.

## Discussion

Intranasal drug abuse appears to be a growing trend. In addition to cocaine, insufflation of heroin and other opioids, stimulants, benzodiazepines, and diet pills has been reported (6-8). Among heroin abusers, insufflation has become a popular method of drug administration, with rates of injection of heroin declining dramatically (9,10). This shift toward intranasal administration may be due in part to increasing awareness of HIV among drug abusers (11,12). 

Xerostomia and septal perforation were discernible in the patient in this case. Erosion of the soft palate and nasal turbinates are more recently reported complications of intranasal prescription narcotic abuse (13). Though palatal perforations and destructive orofacial lesions are uncommonly seen in abusers of drugs other than cocaine, clinicians should be aware that a variety of causative agents and pathologic conditions may be associated with this clinical presentation. The pathogenetic mechanisms responsible for opioid-induced damage, as opposed to cocaine-induced damage, remain unknown. With respect to cocaine-associated lesions, it has been suggested that the local vasoconstrictive effects of cocaine may lead to ischemic necrosis of tissue and ultimately nasal or palatal perforation(14-16). Direct trauma to mucosa anesthetized by cocaine, and irritation by contaminants in the drug, have also been suggested as possible etiologic factors in cocaine-associated lesions (12, 16). Local irritation may further result in stasis of mucocilliary activity, crusting, and bacterial or fungal colonization-ultimately leading to necrosis and ulceration (12,17).

As already mentioned, however, the mechanisms of tissue damage with opioid abuse are unknown. One possible explanation for tissue injury with opioid abuse may lie in the effects of opioids on the immune system (18).Lymphocytes and macrophages are known to possess opioid immunosuppressive effects through the inhibition of cell-mediated immunity, allowing for the development of invasive bacterial or fungal infections in otherwise healthy opioid abusers (13,17,18). In 2 of the 3 previously reported cases of destructive lesions resulting from intranasal prescription narcotic abuse, patients were found to have invasive fungal rhinosinusitis. This finding is somewhat surprising, because invasive fungal rhinosinusitis is typically exclusive to immune-compromised individuals (20). Fungal rhinosinusitis is usually caused by opportunistic pathogens, such as Phyco- mycetes and Aspergillus species, and may result in necrosis of the nasal mucosa. Though invasive fungal rhinosinusitis may be a serious complication of intranasal cocaine abuse, it did not appear to be a factor in the present case.

The relative scarcity of cases of destructive orofacial lesions arising in opioid abusers raises some suspicion that these patients may have used cocaine in the past. In the present case, as well as in previously reported cases, patients denied any prior cocaine use. Toxicology screening, additionally, revealed no evidence of recent cocaine use in our patient. While the possibility of past cocaine use cannot be completely excluded, the potential for opiates or other drugs to produce destructive orofacial lesions should be considered. Finally, we feel a multidisciplinary approach would be the most effective means of managing patients such as the one in this case.

## Conclusion

Patient history and assurance regarding illegal drug consumption and abuse is a key point for fast and accurate diagnosis. Furthermore, each attempt to clarify the drug type and route of consumption leads to prevention of this unwanted complication. This may involve drug counseling and behavior modification in addition to regular chemistry panels to assure discontinuation of the drug habit prior to surgical reconstruction. This approach may increase the likelihood of treatment success. 
